# Complex contraception provision during the COVID-19 pandemic, how did sexual health services fare?

**DOI:** 10.1177/09564624221076616

**Published:** 2022-03-01

**Authors:** Anna Datsenko, Amelia Marriott, Jessica Shaw, Raj Patel, Elizabeth Foley

**Affiliations:** 1 7423 University of Southampton Medical School, Southampton, UK; 2 Solent NHS Trust, Southampton, UK

**Keywords:** Access, contraception, long-acting reversible contraception, sexual health services

## Abstract

**Background:**

This study evaluated whether sexual health services (SHS) across the UK could meet the Faculty of Sexual and Reproductive Health (FSRH) standard for access by being able to offer an appointment for a long-acting reversible contraception (LARC) fitting within 2 weeks of initial contact.

**Methods:**

SHSs offering LARCs were identified using the British Association for Sexual Health and HIV (BASHH) clinic database. During October 2020, all clinics open for more than 1 day a week were contacted by telephone. The researcher posed as a 20-year-old woman in a regular heterosexual relationship who was using condoms and requesting a contraceptive implant. Data collected included the time to wait to appointment and whether clinics offered bridging methods of contraception during any delay in appointment. It was also noted whether a local COVID-19 restriction was in place at the time of the call. The information collected was coded, and data was analysed using chi-square tests in SPSSv27.

**Results:**

Of the 218 contactable clinics, 51.4% (*n* = 112) of clinics offered the patient an appointment within two weeks, and 66.1% (*n* = 144) of clinics could offer appointments within four weeks. 7.3% (*n* = 16) of clinics offered the patient adjunct bridging oral contraception until the time of appointment. Comparing the devolved nations, 11/17 (64.7%) clinics in Scotland, 8/13 (61.5%) clinics in Wales, 0/4 (0.0%) clinics in Northern Ireland and 93/182 (51.1%) clinics in England offered an appointment within two weeks with significant regional variation across England (*p* = .005). No statistically significant difference was demonstrated in access between clinics with or without high-level COVID-19 restrictions (*p* = .056).

**Conclusion:**

The 2-week standard was met in just over half of the occasions, with significant variation across regions across the UK. The development of a national target for access may improve access to LARCs.

## Introduction

Contraception and family planning are one of the most cost-effective investments a country can make for its future.^1^ Financially, contraception saves the NHS £11 for every £1 spent.^2^

Although contraception can be obtained through several sources, this study solely focused on accessing long-acting reversible contraception (LARC) from sexual health services (SHS). Fewer than a fifth of women and men access contraception from community sexual health clinics, but those who do are younger and at greater risk of poor sexual health.^3^

Standards of care regarding access to LARCs have been established by the Faculty of Sexual and Reproductive Health (FSRH) stating that ‘all women choosing LARC methods are offered an appointment to have this fitted within 2 weeks’.^4^ However, as this standard does not specify a national performance indicator for the percentage of women seen within the timeframe, each local authority is able to decide upon this percentage independently. Each authority is able to make their own decisions based on local need with expenses met from their protected ring-faced public grant.^5^ Nonetheless, there is substantial regional difference in how sexual and reproductive healthcare is provided, primarily due to the differences in commissioning in each region.^6^

Furthermore, LARC provision has seen a significant decline in many areas in recent years – with up to 49% of councils reducing the number of sites commissioned to deliver contraceptive services in the past year.^7^ Additionally, the COVID-19 pandemic drastically changed the way the NHS delivered non-essential care. In terms of the contraceptive implant, new fittings were halted during the first national lockdown and women who requested LARCs should have been offered effective methods of oral contraception until restrictions were eased. As the prevalence of COVID-19 decreased, services were able to reinstate the provision of non-urgent contraception that required a face-to-face procedure.^8^

Previous mystery shopper studies have been conducted in the past, evaluating access to sexual health services with researchers posing as ‘patients’. These studies exclusively tested access regarding patients presenting with STIs,^9-11^ with no similar studies conducted on accessing contraception.

With increasing patient numbers, the absence of targets and the impact of the COVID-19 pandemic, there are concerns that accessibility to sexual health services has declined. This study aimed to create a snapshot of complex contraception provision during the COVID-19 pandemic.

## Methods

This was a ‘mystery shopper’ study aiming to evaluate whether sexual health services across the UK could offer a LARC fitting appointment that was within 2 weeks of initial contact during October 2020.

### Data Collection

The British Association of Sexual Health and HIV (BASHH) clinic database was used to identify sexual health services offering LARCs. Clinics that were open less than 2 days a week were excluded from the database. The remaining 241 clinics were telephoned. The database was organised by BASHH region, with each clinic assigned a unique identifying number.

The researcher posed as a 20-year-old woman in a regular heterosexual relationship currently using condoms for contraception and who was requesting a contraceptive implant fitting.

When contacting a clinic, the researcher called up to five times on separate occasions during opening hours. If the phone was not answered on the fifth attempt, the call was classed as ‘unsuccessful’ and the clinic was categorised as could not be reached by telephone. If the call was answered, but the clinic was unable to offer an appointment and advised the researcher to call back the following day – this was also classified as an attempt.

During a successful phone call, the researcher asked for an appointment to have an implant fitted. Whether the 2-week standard was met was calculated from the first ‘successful’ call with exactly 2 weeks later as the cut-off point, to accommodate the clinic’s opening times. If the standard was not met, the time from the call until the ‘patient’ would be seen was calculated.

Calls were made in October 2020 and took place in the morning for consistency.

Additional data was collected in regard to the COVID-19 pandemic, which included the tier the clinic was in or if a local lockdown was in place at the time of the call. Tier three was noted as a local lockdown once the tiers system was introduced. If a clinic could not offer an appointment that was within 2 weeks, how the clinic dealt with the patient was noted – was a bridging method offered?^8^

Once all the necessary data were collected, no actual appointments were booked.

Approval for this study to be undertaken was gained from BASHH and all clinics were notified of this service evaluation through the BASHH clinical governance regional networks.

### Data Analysis

Numerical values were assigned to categorise responses. Each response was assigned a number, allowing the data to be inputted into Excel and SPSS. SPSS v27 was used for statistical analysis and frequency data.

In SPSS Chi^2^ tests were performed as most of the variables were categorical. Tests were done at a 95% significance level where *p* < .05.

Though a standard regarding the percentage of women seen within a specified timeframe has not been outlined by any quality care bodies – certain local authorities have included that ‘>80% of women are offered access to LARC method of choice within two calendar weeks of first contact’^12^ in their reports. Albeit this indicator is not followed in all regions, it was seen as beneficial to compare the data collected against it to identify any regions that may be struggling to uphold timely access.

## Results

All 241 clinics in the UK were successfully contacted once all with the same clinical scenario. 218 clinics were included in data analysis of the 241 that were called. 23 clinics were excluded on a similar premise as before – clinics had changed opening hours and were now open less than 2 days a week and a number of clinics had closed permanently and were not contactable by telephone anymore. Clinics that were temporarily closed and were able to assist the patient were kept in the analysis. GUM clinics in Northern Ireland, Isle of Man and Jersey do not provide contraception and the researcher was referred to sister family planning clinics instead. These clinics were called and used to assess access in those regions.

Out of the 218 clinics, 51.4% (*n* = 112) could offer an appointment that was within 2 weeks of initial contact. 66.1% (*n* = 144) of clinics could offer an appointment that was within 4 weeks.

If the patient was not offered an appointment within 2 weeks, the timings were categorised further to see how clinics were missing the target. Waiting times ranged from just over 2 weeks to a 12-week wait. 8.7% (*n* = 19) of clinics could offer appointments that were over 2 weeks but within 3 weeks and 6.0% (*n* = 13) of clinics could offer appointments that were over 3 weeks but within 4 weeks. 33.0% (*n* = 72) of clinics who could not see the patient within the 2 weeks, could only offer an appointment that was more than 4 weeks after the initial contact. 0.9% (*n* = 2) of clinics had unsuccessful calls and therefore were not contactable by telephone.

Some clinics offered a bridging method of contraception alongside their appointment until the implant could be fitted – 7.3% (*n* = 16) of clinics offered to start a prescription of the progestogen-only-pill.

None of the regions managed to see the patient at all of their clinics within the 2-week timeframe. (See Table 1)

**Table 1.** Timings of appointments offered after initial contact.

	Number of clinics included in the study	Number of clinics excluded from the study	Within (≤) 2 weeks	>2 weeks but within 3 weeks	>3 weeks but within 4 weeks	More than 4 weeks	Offered bridging method of contraception
Overall	218	23	112 (51.4%)	19 (8.7%)	13 (6.0%)	72 (33%)	16 (7.3%)
Country							
England	182	18	93 (51.1%)	15 (8.2%)	12 (6.6%)	60 (33%)	13 (7.1%)
Wales	13	3	8 (61.5%)	2 (15.4%)	0 (0.0%)	3 (23.1%)	2 (15.4%)
Scotland	17	1	11 (64.7%)	1 (5.9%)	0 (0.0%)	5 (29.4%)	0 (0.0%)
Northern Ireland	4	1	0 (0.0%)	0 (0.0%)	1 (25.0%)	3 (75.0%)	1 (25.0%)
Isle of Man and Jersey	2		0 (0.0%)	1 (50.0%)	0 (0.0%)	1 (50.0%)	0 (0.0%)
BASHH branch							
Northern	14	1	8 (57.1%)	2 (14.3%)	3 (21.4%)	1 (7.1%)	0 (0.0%)
North West	20	2	6 (30%)	2 (10%)	3 (15.0%)	9 (45.0%)	3 (15.0%)
Cheshire and Merseyside	12	0	5 (41.7%)	1 (8.3%)	0 (0.0%)	6 (50.0%)	1 (8.3%)
Yorkshire	11	3	7 (63.6%)	1 (9.1%)	0 (0.0%)	3 (27.3%)	0 (0.0%)
Trent	18	2	16 (88.9%)	0 (0.0%)	1 (5.6%)	1 (5.6%)	0 (0.0%)
West Midlands	21	3	4 (19.0%)	3 (14.3%)	0 (0.0%)	14 (66.7%)	3 (14.3%)
East Anglia	9	1	4 (44.4%)	1 (11.1%)	0 (0.0%)	4 (44.4%)	1 (11.1%)
Oxford	11	1	4 (36.4%)	1 (9.1%)	0 (0.0%)	5 (45.5%)	1 (9.1%)
Thames NW	11	1	7 (63.6%)	1 (9.1%)	2 (18.2%)	1 (9.1%)	1 (9.1%)
Thames NE	14	4	7 (50.0%)	1 (7.1%)	0 (0.0%)	6 (42.9%)	1 (7.1%)
Thames SW	6	0	4 (66.7%)	0 (0.0%)	0 (0.0%)	2 (33.3%)	1 (16.7%)
Thames SE	12	0	5 (41.7%)	1 (8.3%)	1 (8.3%)	4 (33.3%)	1 (8.3%)
Wessex	12	0	9 (75.0%)	1 (8.3%)	0 (0.0%)	2 (16.7%)	0 (0.0%)
South West	11	0	7 (63.6%)	0 (0.0%)	2 (18.2%)	2 (18.2%)	0 (0.0%)

Comparing the devolved nations, 64.7% (*n* = 11) clinics in Scotland, 61.5% (*n* = 8) clinics in Wales, 0.0% (*n* = 0) clinics in Northern Ireland and 51.1% (*n* = 93) clinics in England all could offer an appointment within 2 weeks of first contact. Regarding Northern Ireland, none of the clinics could accommodate the patient within 2 weeks.

Focussing on England, seven branches fell below the mean levels of access of 51.1% that other branches exhibited. Branches in England showed a statistically significant difference (*p* = .005).

For further analysis, BASHH branches were compared against an 80% target. Trent was the only region able to meet the target.

Comparing levels of access across local COVID-19 restriction areas, 66.7% (*n* = 22) of clinics which were under a local lockdown could offer an appointment within 2 weeks. Of the clinics that were not under any local COVID-19 restrictions, 48.6% (*n* = 90) of clinics could offer an appointment within 2 weeks. No statistical difference was shown in levels of access between clinics that were under a local lockdown and those that were not (X^2^ = 3.639, df = 1, *p* = .056).

## Discussion

This was a small snapshot study based on one telephone contact by one researcher to UK national sexual health clinics during the COVID-19 pandemic in October 2020, however we feel it is true representation of access to services when requesting an implant in that time period.

The most significant result is that SHSs are collectively not achieving the 2-week standard for access. In just under half of the occasions, clinics failed to offer an appointment that was within 2 weeks. There was marked variation regarding access across the UK, as well as between branches in England. Contraception is a vital service provided by sexual health services with disparities in access affecting vulnerable groups the most. Introducing a national key performance indicator that is upheld by FSRH and other quality care bodies may be beneficial and create a target for sexual health services to maintain.

As previous studies have not been conducted in the past, it is difficult to determine the impact of the COVID-19 pandemic, although local COVID-19 restrictions did not seem to have a significant effect on clinic access.

One of the limitations of this evaluation is that it is a small study with no clinics called more than once, thus the data collected is only a snapshot of clinic access. Additionally, the majority of calls took place within a period of 4 weeks, which may not be reflective of contraceptive provision throughout the entire pandemic. Conducting a large, repetitive service evaluation at this time, however, would have been deemed unethical as health services were already facing an increased pressure. Furthermore, the database used to contact clinics was not created by the current researchers and many changes have occurred since its formation. Lastly, because the data was coded in binary code, raw data calculations were not possible, which would have allowed for more meaningful data analysis.

As sexual health services primarily provide contraception for the younger population who face an already increased risk of poor sexual health, community clinics are in a unique position as they can reach a large proportion of those individuals at risk. Therefore, directing resources and interventions towards sexual health services could help reduce teenage conception rates while simultaneously addressing other sexual health concerns.^13^ Rapid access to different types of contraception must be maintained and regulated – this study illustrates the importance of evaluating how contraception provision in the UK is delivered.

Introducing a standard that outlines the percentage of women who should be offered appointments for a LARC fitting within a specified timeframe by a quality care body could serve as a target for commissioners and clinics to strive towards to reduce disparities in access.
